# Letter to the Editor: a commentary on ‘Safety and efficacy of enhanced recovery after surgery among patients undergoing percutaneous nephrolithotomy: a systematic review and meta-analysis’

**DOI:** 10.1097/JS9.0000000000001561

**Published:** 2024-05-03

**Authors:** Yiqi Huang, Weigang Shen, Meixiang Han, Langping Ling

**Affiliations:** a Department of Nephrology, Shaoxing Second Hospital; b Department of Emergency Internal Medicine, Shaoxing Second Hospital, Shaoxing, Zhejiang, People’s Republic of China

*Dear Editor,*

We reviewed the meta-analysis published by Liu *et al*.^1^ on the safety and effectiveness of Enhanced Recovery After Surgery (ERAS) for patients undergoing percutaneous nephrolithotomy (PCNL) for stone removal. Liang Liu and his team analyzed prognostic indicators for patients after PCNL, demonstrating that ERAS not only ensures the safety of PCNL but also facilitates postoperative recovery for patients. The ERAS protocol is a comprehensive patient care approach that involves nutritional assessment and enhancement, patient education, minimally invasive surgery, and multimodal pain management, tailored to each stage of the patient’s preoperative, intraoperative, and postoperative care based on specific patient needs. ERAS has shown significant advantages in reducing postoperative complications, shortening hospital stays, increasing patient satisfaction, and lowering medical costs^2^. The effective application of the ERAS protocol in PCNL represents a significant advancement in the management of patients undergoing kidney stone surgery.

However, this study faces some limitations that need consideration. Most notably, the meta-analysis only included studies conducted in China. The specific implementation of the ERAS protocols can vary due to hospital policies and geographical locations, and different cultural beliefs and social factors in various regions may also affect patients’ compliance with ERAS recommendations. Including only studies from China does not demonstrate the advantages of ERAS in postoperative management of patients undergoing PCNL in other countries. This could lead to high variability in results and potential outcome bias, limiting the generalizability of the study findings to other populations.

Secondly, the authors stated that when significant heterogeneity was present, they used a random effects model for analysis after excluding clinical heterogeneity. In the analysis of the postoperative hospital stay, a total of 23 studies were included, which exhibited substantial heterogeneity. The authors conducted subgroup analyses based on factors such as sample size and stone location to eliminate clinical heterogeneity. Although this is a robust approach, the possibility of residual confounding due to other unmeasured variables cannot be ignored. The methodological designs vary across different clinical trials, such as differences in baseline criteria for patient inclusion, variations in surgical methods and techniques, and differences in subsequent patient care. The inclusion of a large number of studies inevitably introduces significant clinical heterogeneity, which requires more thorough discussion to address adequately.

Overall, it is clear that future research is needed on the safety and effectiveness of ERAS for patients undergoing PCNL that is more inclusive of different geographical and medical contexts to enhance the external validity of the findings^3^. Furthermore, further studies should focus on standardizing the ERAS protocols across different institutions to better understand which specific components of the protocol are most effective. By addressing these areas, the medical community can better ascertain the full potential of ERAS in improving outcomes for patients undergoing PCNL.

## Ethical approval

Not applicable.

## Consent

Not applicable.

## Sources of funding

None.

## Author contribution

Y.H., W.S., M.H., and L.L.: conceptualization, methodology, validation, formal analysis, investigation, resources, data curation, writing – original draft, writing – review and editing, supervision, and project administration.

## Conflicts of interest disclosure

There are no conflicts of interest.

## Research registration unique identifying number (UIN)

Not applicable.

## Guarantor

Yiqi Huang, Weigang Shen, Meixiang Han, and Langping Ling.

## Data availability statement

Data sharing is not applicable to this article as no new data were created or analyzed in this study.

## Provenance and peer review

Not applicable.
